# MicroRNA-mRNA interactions in a murine model of hyperoxia-induced bronchopulmonary dysplasia

**DOI:** 10.1186/1471-2164-13-204

**Published:** 2012-05-30

**Authors:** Jie Dong, William A Carey, Stuart Abel, Christopher Collura, Guoqian Jiang, Sandra Tomaszek, Shari Sutor, Anja C Roden, Yan W Asmann, Y S Prakash, Dennis A Wigle

**Affiliations:** 1 Division of General Thoracic Surgery, Department of Surgery, Mayo Clinic, Rochester, MN, USA; 2 Division of Neonatal Medicine, Department of Pediatrics, Mayo Clinic, Rochester, MN, USA; 3 Department of Anesthesiology, Mayo Clinic, Rochester, MN, USA; 4 Division of Biomedical Statistics and Informatics, Department of Health Sciences Research, College of Medicine, Mayo Clinic, Rochester, MN, USA; 5 Department of Physiology and Biomedical Engineering, Mayo Clinic, Rochester, MN, USA; 6 Department of Laboratory Medicine and Pathology, Mayo Clinic, Rochester, MN, USA

## Abstract

**Background:**

Bronchopulmonary dysplasia is a chronic lung disease of premature neonates characterized by arrested pulmonary alveolar development. There is increasing evidence that microRNAs (miRNAs) regulate translation of messenger RNAs (mRNAs) during lung organogenesis. The potential role of miRNAs in the pathogenesis of BPD is unclear.

**Results:**

Following exposure of neonatal mice to 80% O_2_ or room air (RA) for either 14 or 29 days, lungs of hyperoxic mice displayed histological changes consistent with BPD. Comprehensive miRNA and mRNA profiling was performed using lung tissue from both O_2_ and RA treated mice, identifying a number of dynamically regulated miRNAs and associated mRNA target genes. Gene ontology enrichment and pathway analysis revealed that hyperoxia modulated genes involved in a variety of lung developmental processes, including cell cycle, cell adhesion, mobility and taxis, inflammation, and angiogenesis. MiR-29 was prominently increased in the lungs of hyperoxic mice, and several predicted mRNA targets of miR-29 were validated with real-time PCR, western blotting and immunohistochemistry. Direct miR-29 targets were further validated in vitro using bronchoalveolar stem cells.

**Conclusion:**

In newborn mice, prolonged hyperoxia induces an arrest of alveolar development similar to that seen in human neonates with BPD. This abnormal lung development is accompanied by significant increases in the levels of multiple miRNAs and corresponding decreases in the levels of predicted mRNA targets, many of which have known or suspected roles in pathways altered in BPD. These data support the hypothesis that dynamic regulation of miRNAs plays a prominent role in the pathophysiology of BPD.

## Background

Bronchopulmonary dysplasia (BPD) was first described in 1967 by Northway *et al.*, who hypothesized that its pathogenesis stemmed from prolonged mechanical ventilation of the surfactant-deficient lung with high concentrations of inspired oxygen (hyperoxia) [1]. Since then, new therapies and advances in ventilatory management have led to the disappearance of the “classic” or “old” BPD lesions described in that landmark study. In the present era, BPD most often affects only extremely premature infants born between 23 and 28 weeks gestation [2]. This “left-shift” in the gestational age of the affected population, during which time critical events in lung development are occurring, has required a profound change in our investigation and understanding of both the histopathology and pathophysiology of BPD.

The developing human lung undergoes a well-described series of morphologic changes, each of which occurs within a discrete period of embryonic and fetal development. It is not until the saccular stage, which begins at 24–26 weeks gestation, that pulmonary alveoli begin to form [3]. The lung of an extremely premature neonate consists of abnormal interstitium, an epithelium with few alveoli-forming secondary crests, an immature pattern of elastin deposition and an incompletely developed vasculature. Interestingly, these histological findings persist in the lungs of neonates who require long-term mechanical ventilation after delivery at an extremely premature gestational age. This so-called arrest of alveolar development defines the modern-day or “new” BPD [4].

Currently little is known about the molecular and cellular basis of BPD [2,4,5]. This may be due, in part, to the complexity and prohibitive expense of traditional animal models of newborn lung injury, such as prolonged mechanical ventilation of premature baboons or lambs [4,6]. In this regard, the murine hyperoxia model may provide an ideal alternative to study the pathologic arrest of alveolar development in BPD. Recent research has shown that exposing newborn mice to high concentrations of ambient oxygen recapitulates the histopathology of BPD [7-9]. This is an important finding, as the lungs of mice born naturally at term have reached the same developmental stage as those of the extremely premature human neonate [7,10]. Furthermore, current technologies have greatly facilitated generation and availability of genetically engineered mouse strains that will allow dissection of molecular pathways in BPD.

Alveolar development is a complex process involving multiple mechanisms relating to cell cycle, cell adhesion, mobility and taxis, and angiogenesis. Recent work by our group has demonstrated the dynamic regulation of microRNAs (miRNAs) during lung organogenesis [11]. MiRNAs are a class of small non-coding RNA that regulate gene expression either by inhibiting protein translation or by cleavage of mRNA targets based on the pairing of miRNAs and their mRNA target binding sites [12]. Individual miRNA may target multiple mRNAs, and individual mRNA may contain sequences complementary to multiple miRNA family members [13,14]. It is estimated that miRNAs may be responsible for regulating the expression of nearly one-third of the human genome [15]. MiRNAs are known to play multiple roles in organ development, carcinogenesis, and immune responses [16,17], and have been implicated in many critical cellular processes, including apoptosis, proliferation, and differentiation [18]. Despite the identification of more than 800 mature human miRNAs and 700 mouse miRNAs, much remains to be discovered about their functional targets and biologic role.

In the current study, we explored the regulation of miRNAs and corresponding target mRNAs during the arrest of alveolar development prominent in a murine model of BPD. We provide evidence that dynamic regulation of miRNAs may play a prominent role in the pathophysiology of BPD.

## Results

### Prolonged hyperoxia impairs somatic growth and lung development in neonatalmice

Each of the experimental groups were comprised of 11 mice, all of which survived their respective exposure periods. The 14 and 29 day time points were chosen to capture a cross section of hyperoxic exposure during alveolar development in the mouse. Among mice exposed to prolonged hyperoxia, both somatic growth and lung development were reduced in comparison to room air-exposed controls. The body weight of O_2_ mice was slightly lower than that of normoxic mice after 14 days of exposure, but was markedly lower after 29 days of exposure (not shown).

**Figure 1:**
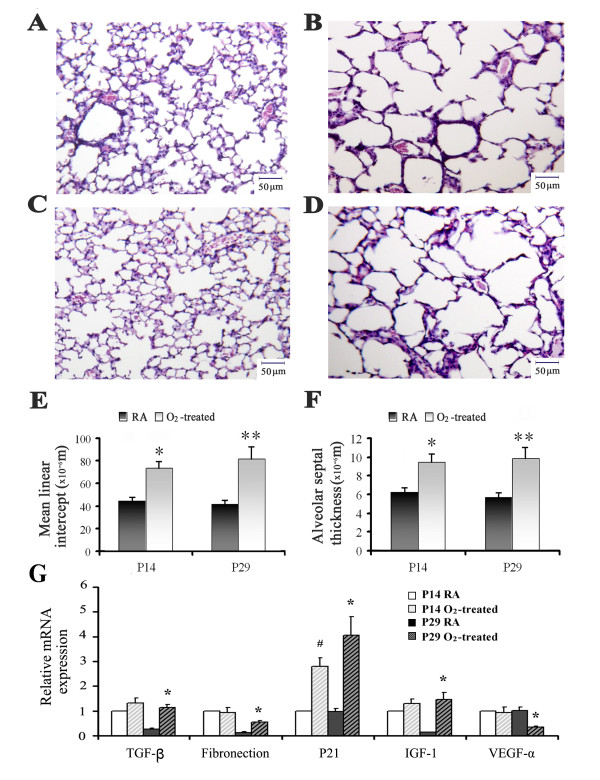
**Effects of hyperoxia on neonatal mouse lung.** Inflated lungs were paraffin embedded and five-micrometer tissue sections were stained with hematoxylin and eosin (H&E). All pictures were taken at 200 x magnification. Calibration bar is 50 μm. **(A)** Exposure to room air at postnatal day 14 **(B)** Exposure to hyperoxia at postnatal day 14 **(C)** Exposure to room air at postnatal day 29 **(D)** Exposure to hyperoxia at postnatal day 29 **(E)** Mean linear intercept **(F)** Alveolar septal thickness (n = 11 mice/group; mean ± SE, * p < 0.05 P14-O_2_ vs. P14-RA, ** p < 0.05 P29-O_2_ vs. P29-RA). **(G)** Relative mRNA expression of several biomarkers of BPD by RT-PCR comparing mouse lung exposed to room air with hyperoxia (n = 3, mean ± SE, # p < 0.05 vs. P14 room air controls, * p < 0.05 vs. P29 room air controls).

Prolonged hyperoxia caused substantial alterations in lung morphology. After 14 days of exposure, the lungs of O_2_ mice contained fewer alveoli than those of RA mice, with generalized enlargement of alveolar airspaces. These histological changes were even more pronounced after 29 days of exposure, with little or no morphologic maturation seen in the lungs of O_2_ mice. On average, compared with normoxic mice, Mean linear intercept (MLI; which represents the average alveolar diameter) was increased by 66% and 93% after 14 and 29 days of hyperoxic exposure, respectively. Alveolar septal thickness (AST) was increased by 50% and 73% for the same groups, respectively (Figure 1A-F). Gene expressions of several well-known BPD biomarkers were measured by Real-time PCR. Transforming growth factor (TGF)-β signaling is involved in pulmonary fibrosis and inhibition of branching morphogenesis in lung development [19]. TGF-β mRNA was increased 4.2 fold in hyperoxia-induced BPD mice. Fibronectin 1, a component of the extracellular matrix [20], was also increased 4.5 fold at day 29. Vascular endothelial growth factor (VEGF)-α is an endothelial cell mitogen that regulates endothelial cell differentiation and angiogenesis [21], with expression decreased by 65% after hyperoxia. Insulin-like growth factor (IGF)-1 was dramatically increased by 10.2 fold in the lung of O_2_ treated adult mice. IGF has been shown to be a strong profibrogenic mediator, acting as a potent mitogen and stimulator of collagen synthesis [22]. The expression of p21 was increased 6.2 fold, indicating hyperoxia can cause proliferation arrest of cells in the lung [23] (Figure 1G). Taken together, these biomarker changes, accompanied by pulmonary morphological changes, are consistent with development of human BPD.

### Prolonged hyperoxia alters mRNA expression in the neonatal mouse lung

To investigate genes dynamically regulated as a consequence of hyperoxia, mRNA expression profiling was performed using the Affymetrix GeneChip Mouse Genome 430 2.0 array. In total, 12 samples were used for mRNA profiling, 2 samples for each normoxia group at time points of P1, P14, and P29; and 3 samples for each hyperoxia-treated group at time points P14 and P29. The log_2_ expression values of 45,037 mRNA probe sets were obtained after normalization using Partek Genomics Suite Software. When the cut-off value of fold change was set as > |2|, and the p-value for ANOVA analysis was set as p < 0.05 and further adjusted by False Discovery Rate (FDR < 0.05) to p < 0.018, 1904 significant probes were identified for comparison Group A (P29-RA vs. P14-RA), 1386 probes for comparison Group B (P14-O_2_ vs. P14-RA) and 1961 probes for comparison Group C (P29-O_2_ vs. P29-RA), suggesting discrete temporal patterns of gene expression over the course of lung development and hyperoxia-induced injury. In total, 2714 unique mRNAs were identified as hyperoxia-responsive genes from Group B and Group C.

**Figure 2:**
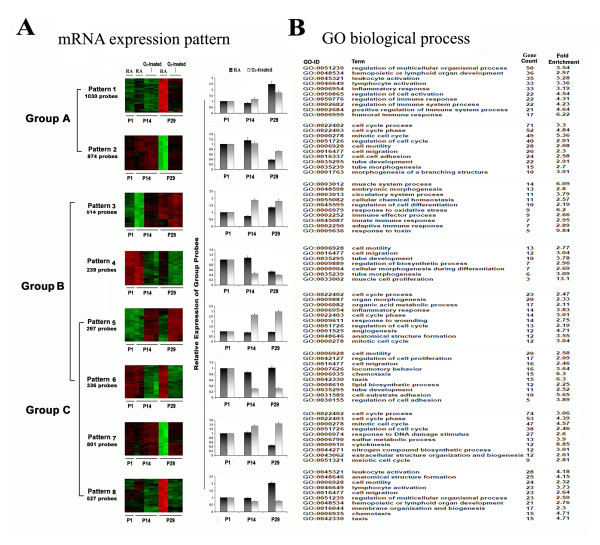
**Expression patterns of hyperoxia-responsive mRNAs and functional analysis.** Total RNA was isolated and RNA quality was confirmed. In total, 12 samples were used for analysis of mRNA expression; 2 samples for each normoxia group at time points of P1, P14, and P29; and 3 samples for each hyperoxia-treated group at time points P14 and P29. Gene expression profiling was performed using the Affymetrix GeneChip Mouse Genome 430 2.0 Array. Expression patterns of hyperoxia-responsive mRNAs and corresponding gene ontology mapping were performed. **(A)** 8 expression patterns in 3 groups represented by color heat maps and bar graphs (x axis indicating the 3 time points, y axis indicating the relative expression value of group probes). Red represents increased expression and green represents decreased expression in the depicted heat maps. The relative expression values in each time point are the ratios normalized against those in the P1 group. Group A: P29-RA vs P14-RA, Group B: P14-O_2_- treated vs P14-RA, Group C: P29-O_2_-treated vs P29-RA **(B)** Analysis of enrichment for GO biological process categories for 8 expression patterns. Top 10 GO terms (ranked by count number) with p < 0.05 are listed.

For each comparison group, we initially divided the significant probes into two categories based on the positive- and negative-fold changes in transcript levels. However, as shown in Figure 2A, there was significant overlap between the expression patterns of Groups B and C, such that probes for these groups were divided into six patterns: Pattern 3 and 4 were unique to Group B; Pattern 5 and 6 were common to both Groups B and C; and Pattern 7 and 8 were unique to Group C (Figure 2A). We infer that the overlapping clusters may contain transcripts of the genes most relevant to the pathophysiology of BPD.Through the biological function analysis of 8 distinct mRNA expression patterns using the online biological classification tool DAVID, the top 10 gene ontology (GO) terms ranked by gene count number with p < 0.05 were identified (Figure 2B). For Group A, representing normal lung development across two time points, genes with increased expression (Pattern 1) were enriched with immune and inflammatory response genes. Those with decreased expression values (Pattern 2) were enriched for the terms cell motility, adhesion and cell cycle. For both of Group B and C which represent the hyperoxia-responsive genes, the genes with increased expression (Pattern 3, 5 and 7) were enriched for immune response and cell cycle, while the genes with decreased expression (Pattern 4, 6 and 8) were enriched in functions related with cell motility and taxis.

### Prolonged hyperoxia alters miRNA expression in the neonatal mouse lung

To investigate dynamically regulated miRNAs as a consequence of hyperoxia, miRNA expression profiling was performed. We used the Taqman Rodent MicroRNA array, which assays 521 mature mouse miRNAs based on real-time PCR methodology. In total, 12 samples were used for miRNA profiling, 2 samples for each normoxia group at time points of P1, P14, and P29; and 3 samples for each hyperoxia-treated group at time points P14 and P29. In all, 143 miRNAs were detected and their values were normalized using small nuclear U6 RNA as an internal control. When the cut-off value of fold change was set as > |4|, and the p-value was set as p < 0.05 and further adjusted by FDR (FDR < 0.05) to p < 0.027, we found 68 species which were dynamically regulated. 7 of these occurred in Group A, 31 in Group B and 64 in Group C.

**Figure 3:**
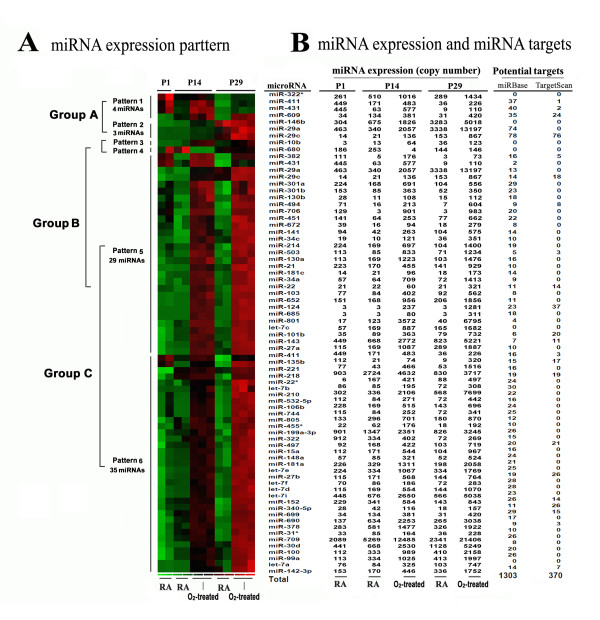
**Expression patterns of hyperoxia-responsive miRNA and miRNA targets.** Total RNA was isolated and RNA quality confirmed. In total, 12 samples were used for miRNA profiling, 2 samples for each normoxia group at time points of P1, P14, and P29; and 3 samples for each hyperoxia-treated group at time points P14 and P29. MiRNA expression profiling was performed using the RT-PCR based Taqman Rodent MicroRNA array measuring 521 mature mouse miRNAs. Raw miRNA array data were analyzed using RQ manager software. **(A)** 6 expression patterns in 3 groups represented by color heat maps. **(B)** MiRNA expression and prediction of miRNA targets. Average miRNA expression is shown as copy number. Predicted targets indicate miRNA targets identified by overlapping computationally predicted mRNA targets with the mRNAs having opposite expression patterns with their corresponding individual miRNAs in our data. When two mature miRNAs originate from opposite arms of the same pre- miRNA, they are denoted with a -3p or -5p suffix. When relative expression levels are known, an asterisk following the name indicates a miRNA expressed at low levels relative to the miRNA in the opposite arm of a hairpin, for example, miR-322 and miR-322* would share a pre-miRNA hairpin, but more miR-322 would be found in the cell [24].

For each comparison group, we initially divided the significant miRNAs into two categories based on the positive- and negative-fold changes in transcript levels. In Group A, the expression values of four miRNA were decreased (Pattern 1; miR-322*, miR-411, miR-431, miR-609) and three were increased (Pattern 2; miR-146b, miR-29a, miR-29c). As was the case with mRNA transcription, there was significant overlap between the miRNA expression patterns of Groups B and C, such that miRNAs for these groups were divided into four patterns (Figure 3A). Pattern 3 and 4 were unique to Group B (increased miR-10b and decreased miR-680, respectively); Pattern 5 was common to both Groups B and C and contained 29 miRNAs whose expression was increased; and Pattern 6 was comprised of 35 Group C miRNAs whose expression was likewise increased.

Among the 7 miRNAs dynamically regulated over the course of normal lung development (Group A), 5 of these miRNAs (miR-411, miR-431, miR-699, miR-29a and miR-29c) were up-regulated by oxygen exposure, suggesting that prolonged hyperoxia alters the expression of miRNAs utilized during normal lung development. Surprisingly, within 66 hyperoxia-responsive miRNAs, all but one was up-regulated. The top 5 fold-increased miRNA were miR-124 (450-fold), miR-706 (345-fold), miR-801 (171-fold), miR-685 (109-fold) and miR- 494 (93-fold). The top 5 high expression value miRNAs were miR-709, miR-29a, miR-210, miR-810 and miR-143; miR-709 was the most highly expressed with a copy number of 23,676 per cell.

### MiRNAs modulate mRNA expression in the neonatal mouse lung exposed to hyperoxia

Given that miRNAs are known to negatively modulate the level of “target” mRNAs, we considered individual increased miRNAs as those potentially having mRNA targets with decreased expression in our data. When combined with computational prediction, simultaneous profiling of miRNA and mRNA levels can be a strategy for the identification of putative functional miRNA target mRNAs [25,26]. Two time points provide further information that could readily be missed in a cross-sectional study based on a single time point [27]. For this analysis, mRNA transcripts whose expression was reduced in the O_2_ groups were considered as potential targets for every individual miRNA whose expression was simultaneously increased. We then cross-referenced these potential mRNA targets against the computationally predicted targets of the 66 hyperoxia-responsive miRNAs from miRBase and TargetScan. In all, we identified 1303 miRNA-mRNA probe pairs, involving 581 unique mRNAs from miRBase, and 370 miRNA-mRNA probe pairs which included 268 unique mRNAs from TargetScan. In total, 152 mRNAs overlapped as targets between the two databases (Figure 3B).

### MiR-29 modulates development in the neonatal mouse lung exposed to hyperoxia through Ntrk2

**Figure 4:**
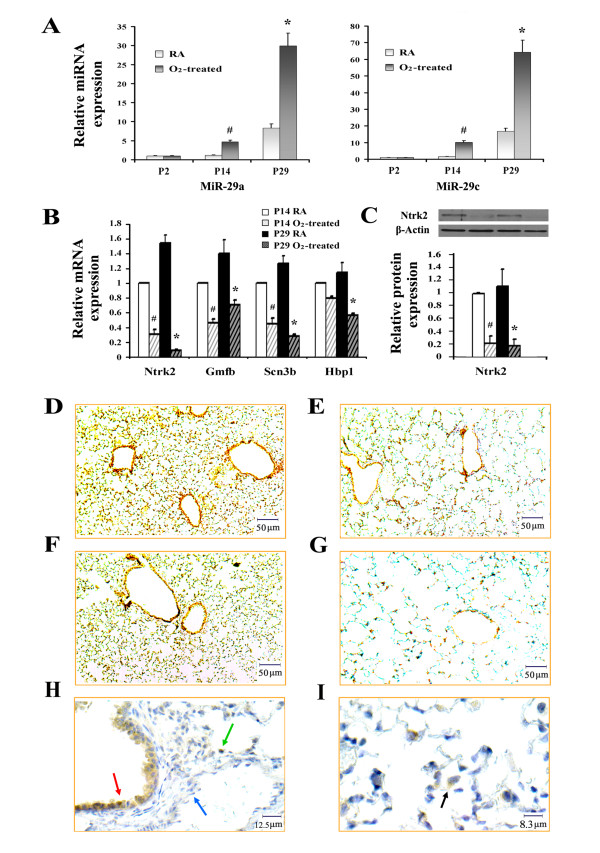
**Effects of hyperoxia on the expression of miR-29 and predicted mRNA targets.** (**A**) Relative expression of miR-29a and miR-29c (n = 3, mean ± SE, # p < 0.05 vs. P14 room air controls, * p < 0.05 vs. P29 room air controls). (**B**) Relative mRNA expression by RT-PCR comparing mouse lung exposed to room air with hyperoxia (n = 3, mean ± SE, # p < 0.05 vs. P14 room air controls, * p < 0.05 vs. P29 room air controls). (**C**) Nrtk2 protein expression measured by Western blot analysis using β-actin as the loading control (n = 3, mean ± SE, # p < 0.05 vs. P14 room air controls, * p < 0.05 vs. P29 room air controls). Protein expression of Nrtk2 in the lung of P14-RA (**D**), P14-O_2_-treated (**E**), P29-RA (**F**) and P29-O_2_-treated mice (**G**). Nrtk2 immunoreactivity was detected in brown. (**H**) and (**I**) Immunohistochemical localization of Nrtk2 in P29-RA lung. Red arrow indicates bronchiolar epithelial cells, green arrow indicates type II pneumocyte, blue indicates endothelial cells and black indicates macrophage.

Expression of the MiR-29 family is increased during normal mouse lung development, and is known to play an important role in the pathogenesis of lung diseases such as pulmonary fibrosis [28,29]. Analysis of normal lung has shown the presence of miR-29 in subsets of cells in the alveolar wall and entrance to the alveolar duct [28]. In our miRNA array and real-time PCR data, miR-29a and miR-29c were increased during normal lung development, and expression was dramatically increased with the induction of hyperoxia (Figure 4A). Based on computational predictions from both miRBase and TargetScan, there are a total of 25 predicted target mRNAs for miR-29 within our data displaying decreased expression. We evaluated a number of these potential targets through both quantitative real-time PCR and immunohistochemistry. We found neurotrophic tyrosine kinase receptor type 2 (Ntrk2), glia maturation factor beta (Gmfb), voltage-gated sodium channel (Scn3b) and high mobility group box transcription factor 1 (Hbp1) expression all decreased significantly as identified in the array data (Figure 4B). In particular, Ntrk2 mRNA and protein expression in the hyperoxic lung was dramatically decreased in comparison to mouse lung exposed to room air. By immunohistochemistry, immunoreactivity and density for Ntrk2 was also significantly decreased in the lung exposed to hyperoxia (Figure 4D-G). We observed expression of Ntrk2 distributed around small bronchioles and scattered within the alveolar wall in normal lung, consistent with previous reports that Ntrk2 is expressed in epithelial cells of bronchioles, type II pneumocytes and macrophages (Figure 4H-I) [30].

### MiR-29 modulates predicted target mRNAs in mouse bronchoalveolar stem cells (BASCs)

**Figure 5:**
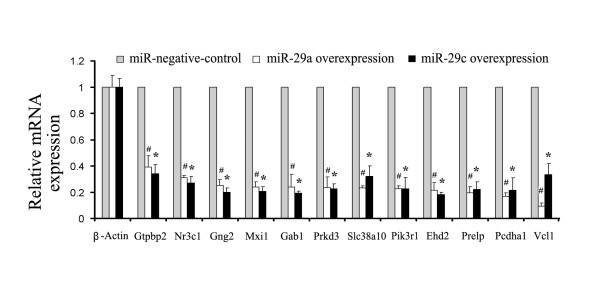
**Validation of predicted miR-29 targets.** Total mRNA were isolated after 24 hours transfection of BASC cells with miR-29a mimic, miR29c mimic and non-specific miRNA-negative control. MiR-29a and miR-29c overexpression were confirmed by real-time PCR (Additional file 1: Figure S1). Relative gene expression of predicted target mRNAs decreased more than 50% in BASC cells are shown (n = 3, mean ± SE, # p < 0.05 miR-29a vs. miR-negative control, * p < 0.05 miR-29c vs. miR-negative control). Results are from real-time PCR with β-Actin as a housekeeping control.

To investigate direct targets of miR-29, we identified candidate mRNAs by overlapping computational prediction with opposite expression patterns in our miRNA and mRNA data. MiR-29 was then overexpressed in vitro by transfection of miR-29a and miR-29c mimics into bronchoalveolar stem cells (BASC), isolated and cultured in our laboratory as previously described [31]. BASC cells are located in the bronchoalveolar duct junction, populate the Type I and Type II pneumocyte and Clara cell niches, and are thought to regenerate both bronchiolar and alveolar epithelium during homeostatic turnover and in response to injury [32]. After 24 hours following transfection, mRNAs were isolated and miR-29 expression measured by RT- PCR. We observed a limited degree of coincident expression increases of either miR-29a or miR-29c after transfection of respective miR-29a or miR-29c mimics (Additional file 1: Figure S1). This phenomenon can likely be explained by the one base pair difference in their respective sequence [33]. After overexpression of miR-29 was confirmed, we examined mRNA expression for 23 of 25 predicted target genes, excluding 2 with no defined function. All mRNAs except Gmfb, were decreased in expression (Additional file 2: Table S5), including 12 of 23 target genes significantly decreased more than 50% (Figure 5). Ntrk2 mRNA expression was decreased by 43% and 44% (n = 3, p < 0.05) after transfection of miR-29a and miR-29c respectively.

### Hyperoxia alters the expression of genes which regulate multiple biological processes

Given the large number of dynamically expressed genes in the hyperoxia groups, we sought to organize the expression data on the basis of biological processes. We employed DAVID to classify the significant hyperoxia-responsive mRNAs into functionally related clusters. In all, 2714 mRNA probes from Groups B and C were entered as input. Classification Stringency was set as “Medium”, using criteria p < 0.05, FDR < 0.05. We identified 6 functional clusters among these transcripts (Enrichment Score > 6.89): cell cycle; cell adhesion; cell mobility and migration; cell taxis and response to external stimulus; vascular development and angiogenesis; and stress and inflammation (Additional file 3: Table S1). Within these 6 functions, a total of 499 genes were identified to be involved. Additional file 4: Table S2 contains a list of these genes, including title; symbol ID; biological function; fold-change direction; time change point and, in the case of genes with decreased expression, the corresponding miRNA predicted to regulate the transcript.

**Figure 6:**
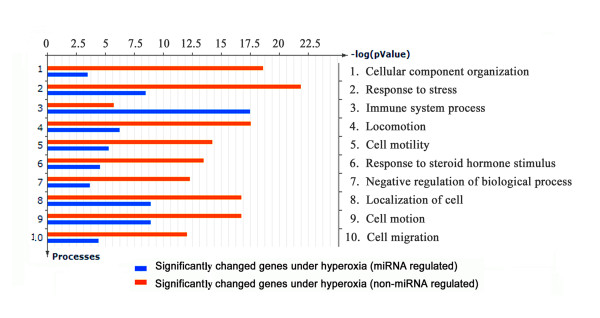
**Biologic function of miRNA-regulated genes under hyperoxia. **Gene ontology process term enrichment between miRNA-regulated and non-miRNA-regulated genes under hyperoxia was compared using the MetaCore enrichment analysis tool. Top 10 GO process enrichment terms were sorted by the “differentially affected maps”, and sorted in decreasing order of the standard deviation of the -log (p-Value) between the two groups. The bar in blue colour represents the GO process term enrichment for the group of miRNA-regulated genes and the bar in red colour for the group of non-miRNA-regulated genes.

Individual miRNAs may target multiple mRNAs, and individual genes may be regulated by a number of miRNAs or multiple members of the same miRNA family. Groups of miRNAs which change in specific biological conditions have predicted propensity to target genes with relative functions which can provide insight into the biological roles of miRNAs [34,35]. To explore if there was a difference in biological function between miRNA-regulated hyperoxia- responsive genes and non-miRNA-regulated hyperoxia-responsive genes, we compared the GO process term enrichment between the two groups using the MetaCore enrichment analysis tool. Figure 6 shows the distribution histogram of the most enriched 10 GO process terms as sorted by “differentially affected maps,” which lists terms in decreasing order of the standard deviation of the -log (p-Value) between the two groups. Thus, the top terms represented by this histogram would have the largest absolute difference in their strip lengths, indicating the GO process term enrichment of the two groups is significantly different. As shown in Figure 6, miRNA-regulated genes (blue strip) more often were enriched in the term immune system process than were non- miRNA-regulated genes (red strip). Genes not regulated by miRNAs were more often enriched in all other functional categories, most notably response to stress, locomotion*, and* cell motility.

### Overlap of dynamically regulated miRNAs in normal lung alveolar development and hyperoxia-induced BPD

MiRNAs play an important role during mouse lung development. Recent work by our group demonstrated that 117 significant miRNAs were dynamically regulated during mouse lung organogenesis from 7 time points compassing all recognized stages of lung development beginning at embryonic day 12 and continuing to adulthood [11]. Among the 66 miRNAs identified from the O_2_-treated group in this study, 33 miRNAs overlapped with the 117 miRNA identified during lung organogenesis from our previous study, suggesting that prolonged hyperoxia may interrupt the dynamic expression of miRNAs involved in normal lung development (Additional file 5: Table S3). This observation provides further evidence for the potential characterization of BPD as an arrest of normal pulmonary alveolar development.

## Discussion

The network of miRNA-mRNA interactions in animal models of BPD has not been described and remains poorly understood. We explored the dynamic regulation of miRNAs and mRNAs over two time points of lung development under 80% O_2_-treatment through genome-wide mRNA expression and real-time PCR based miRNA profiling. From our miRNA array profile, strikingly, 65 of 66 dynamically regulated miRNAs were increased in expression; only one was decreased, suggesting that increased miRNA expression may contribute to the down-regulation of key genes necessary for normal alveolar development to proceed. Given that miRNAs regulate the cleavage of targeted mRNAs, we focused on negatively regulated hyperoxia-responsive genes to identify potential targets of miRNAs. After combining these potential targets with computational prediction, 581 unique mRNAs were identified as targets of miRNAs using miRBase, suggesting that miRNAs regulated 53% of all repressed hyperoxia-regulated genes in our dataset. While these correlations do not infer direct causality, they nonetheless suggest a significant contribution of miRNA-based regulation of gene expression to the alterations observed in this mouse model of BPD. By GO biological process term enrichment analysis, in contrast with non-miRNA-regulated genes, miRNA-regulated genes were particularly enriched for the GO term “immune system process”. Immune system dysfunction has been postulated as a contributing factor in the pathophysiology of BPD, as imbalances of “pro-inflammatory" and "anti-inflammatory" cytokines and impairment in the transition from the innate immune response mediated by neutrophils to the adaptive immune response mediated by T lymphocytes has been described [36]. We validated a number of our microarray-based observations using real-time PCR and immunohistochemistry. Interestingly, we observed increased expression of miR29c in conjunction with decreased expression of its predicted mRNA target Ntrk2. In normal lung, Ntrk2 protein by IHC was found to be located within the alveolar wall and around small bronchioles, consistent with previous reports [30]. In response to hyperoxia, we observed a similar distribution but dramatically decreased staining and protein expression for Ntrk2. Ntrk2 is essential for the normal development of the lung, as transgenic deletion results in thinned out bronchial epithelium and larger alveolar air spaces, a phenotype strikingly similar to that observed in our mouse BPD model [30]. Downregulated Ntrk2 expression, regulated by miR-29c, may play an important role for the development of hyperoxia-induced bronchopulmonary dysplasia and clearly represents an avenue for further research. A number of genes identified as predicted miR-29 targets were also validated in a miR-29 overexpression model using BASC cells. For this specific cell type in vitro, one of many in the developing lung, miR-29 was shown to directly modulate expression of predicted target mRNAs. The influence of specific lung cell types on predicted miRNA-mRNA interactions relevant to the pathophysiology of BPD awaits further investigation.

Through GO biological process term enrichment analysis, we found hyperoxia-responsive genes mainly involved in six biological functional groups including cell cycle, motility, adhesion, taxis, angiogenesis and inflammation. Cell cycle plays an essential role for cell proliferation and further lung organogenesis. p21, a critical inhibitor of the cell cycle, is up-regulated in hyperoxia-induced mouse and baboon models of BPD and oxidative stress [37,38]. In our study, p21 and other cell cycle inhibitors, including many Cyclin (Ccn) and Cell division cycle (Cdc) family members, were ignificantly up-regulated. Several growth factor expressions were found to be up-regulated in our data, such as TGF-β2, IGF, platelet-derived growth factor (PDGF) and connective tissue growth factor (CTGF), TGF-β signaling can negatively regulate the branching and septation phases of lung development. Adenoviral-mediated transfer of TGF-β o the neonatal rat lung or overexpression of TGF- β between postnatal day 7 and day 14 in the mouse can induce histological changes analogous to those seen in BPD [19,39]. VEGF and platelet-endothelial cell adhesion molecule-1 PECAM1) have been reported to promote pulmonary vascular development [40,41], and down-regulated expression of VEGF-A and PECAM1 have been reported in the neonatal lung with severe BPD [42]. In our data, VEGF, ECAM1 and numerous genes involved in angiogenesis were down-regulated, as well as angiogenic signaling mediators PI3K, p85 and Akt-1. Among these genes, our data suggest that the expression of PECAM1 was regulated by let-7i, miR-322 and miR-497, and the expression of VE-cadherin and β-cadherin regulated by miR-27a.In summary, we describe the dynamic regulation of gene and miRNA expression in a hyperoxia-induced model of BPD in the mouse. The abnormal alveolar development observed is accompanied by significant increases in the levels of multiple miRNAs and corresponding decreases in the levels of predicted mRNA targets, many of which have known or uspected roles in pathways altered in BPD. The data also suggest that BPD is a multifactorial disorder characterized by multiple pathway defects as opposed to one overriding abnormality. What the inciting triggers are for the initiation of BPD, how these abnormalities are maintained, and how this relates to normal alveolar development will clearly require a systems-based approach using genetically tractable model organisms such as the mouse. The results overall suggest that the dynamic regulation of miRNAs and the subsequent downstream target effects on mRNA and protein expression may play a prominent role in the pathophysiology of BPD.

## Conclusions

In newborn mice, prolonged hyperoxia induces an arrest of alveolar development similar to that seen in human neonates with BPD. This abnormal lung development is accompanied by significant increases in the levels of multiple miRNAs and corresponding decreases in the levels of predicted mRNA targets, many of which have known or suspected roles in pathways altered in BPD. These data support the hypothesis that dynamic regulation of miRNAs plays a prominent role in the pathophysiology of BPD.

## Methods

### Oxygen exposure protocol

Approval of the study protocol was obtained from the Mayo Clinic Institutional Animal Care and Use Committee with protocol number A41708. All experiments were carried out according to the provisions of the Animal Welfare Act, the tenets of the Public Health Service Policy on Humane Care and Use of Laboratory Animals, and the principles of the National Institutes of Health Guide for the Care and Use of Laboratory Animals. Time-pregnant ICR mice were maintained on an *ad libitum* diet of standard chow and water. Within 12 hours of birth, ups were pooled and then randomized into four experimental groups. Mice were exposed to hyperoxic (80% oxygen) or room air (21% oxygen) conditions for either 14 or 29 days. The hyperoxic environment was established by ventilating a Plexiglas chamber (approximately 100 × 50 × 50 cm) with 3.5 L/min pure oxygen. Oxygen levels were continuously monitored with a Miniox III monitor, and carbon dioxide levels were spot-checked at the outset to ensure normal ambient carbon dioxide levels within the chamber. Room air-exposed mice were housed in standard cages. Nursing dams were rotated between exposure groups every 24 hours to prevent oxygen toxicity in the dams and to mitigate maternal effects between the experimental groups.

### Preparation of lung tissue

Mice were exposed to 80% or 21% O_2_ for 14 days or 29 days. Body weights were recorded and mice were sacrificed by CO_2_ narcosis at the end of the exposure period. In half of the animals within each group, lungs were immediately inflated to 20 cm H_2_O pressure with 10% buffered formalin. After overnight fixation, the inflated lungs were paraffin embedded and 5 μm tissue sections were stained with hematoxylin and eosin (H&E). Alveolar morphology was assessed by measuring mean linear intercept (MLI) and alveolar septal thickness (AST) on H&Estained slides using standard imaging techniques [43]. For the remaining animals, lungs were removed and immediately frozen and stored in liquid nitrogen for further use.

### MicroRNA and gene expression analysis

Total RNA was isolated using Trizol Reagent (Invitrogen) and the mir VanaTM miRNA Isolation kit (Ambion) for mRNA and miRNA microarray analysis according to manufacturer’s instructions. RNA quality and integrity were confirmed by denaturing gel electrophoresis. In total, 12 samples were used for mRNA and miRNA profiling respectively; 2 samples for each normoxia group at time points of P1, P14, and P29; and 3 samples for each hyperoxia-treated group at time points P14 and P29. Messenger RNA expression profiling was performed using the Affymetrix GeneChip Mouse Genome 430 2.0 Array containing probes to query more than 45,000 transcripts. The reverse transcription, labeling and hybridization of mRNA were performed in the Mayo Clinic Advanced Genomic Technology Center.MiRNA expression profiling was performed using the Taqman Rodent MicroRNA Array Card A and Card B (Applied Biosystems) containing all 521 mature mouse miRNA in miRBase 10.1 http://microrna.sanger.ac.uk based on real-time PCR methodology. In brief, miRNA was reverse transcribed to cDNA using the Megaplex TM RT Rodent Primers Pool and the TaqMan MicroRNA Reverse Transcription kit. Quantitative 384 well TaqMan Low Density Array real- time PCR was run on the ABI PRISM 7900 System using TaqMan Universal PCR Master Mix. Raw miRNA array data were analyzed using the RQ manager software on the ABI System. All undetectable data or data with C_T_ values >35 were treated as 35 according to standard protocols [44]. For each miRNA, a normalized C_T_ value (ΔC_T_) was calculated by comparing each miRNA value to that of small nuclear U6 RNA, a common internal control in microRNA arrays. The copy number per cell of each miRNA then was determined, assuming that each cell contains 30 pg total RNA and using the accepted formula: 10^(40-CT)/3.34^/22 [45].

All microarray data have been submitted to the Gene Expression Omnibus (GEO) database with accession number GSE25286 for mRNA expression array and GSE25290 for miRNA array.

### Data processing and analysis

Both the miRNA and the mRNA array data were analyzed using the Partek Genomics Suite 6.4 software. For mRNA expression data, Affymetrix CEL files were imported. The data were normalized with the Robust Multichip Average Algorithm [46] and converted to log_2_ values. For miRNA data, ΔC_T_ values were directly imported as log_2_ values. Greater ΔC_T_ values were interpreted as lower miRNA expression values. Logarithmic data were used for further statistical analysis. After eliminating genes with low expression levels in all time points, fold change and the False Discovery Rate (FDR) adjusted p-values derived from two-way ANOVA analysis were used to filter out significantly changed miRNA and mRNA probes. The False Discovery Rate (FDR) of a set of predictions is the expected percent of false predictions in the full set of predictions.Hyperoxia-responsive miRNA and mRNA probes were divided into 3 different groups according to the interactions between 2 time points (P14 vs. P29) and two types of treatment (O_2_ vs RA). Group A compared Day 14 and Day 29 RA mice; Group B compared O_2_ and RA mice at Day 14; and Group C compared O_2_ and RA mice at Day 29. The comparison of O_2_-treated mice at 14 and 29 days revealed all mRNA and miRNA expression changes to be present in either groups B or C; as a consequence we did not analyze this group further. Within each group, data was divided into positive- and negative-fold change values that were visualized on the heat map produced by dChip software (http://www.dchip.org). We assumed that an individual upregulated miRNA would have potential mRNA targets with downregulated expression in the same group. Overlapping these potential targets with the computational mRNA targets of each miRNA retrieved from the miRBase (version 5) and TargetScanMouse (5.1) databases, we identified a collection of direct mRNA targets for each miRNA. The software package g: Profiler was used to convert Transcript IDs from miRBase into Affymetrix mouse 430 probe set IDs [47].

### Gene ontology and pathway analysis

Gene Ontology (GO) provides a structured ontology of defined terms representing putative functional properties of specific gene products. Using whole probe sets from the Affymetrix mouse 430 2.0 array as a reference gene list, GO enrichment analysis was performed using the Database for Annotation, Visualization and Integrated Discovery (DAVID, National Institutes of Health) [48]. Functional differences between miRNA-regulated or non-miRNA-regulated genes were investigated using the Compare Experiments workflow tool (MetaCore).

### Quantitative real-time PCR

After reverse-transcription (Invitrogen), absolute quantitative real-time was performed using Brilliant SYBR Green QPCR Master Mix (Stratagene) and the PRISM 7900 system (Applied Biosystems) according to the manufacturer’s instructions. After PCR, melting curves were constructed to ensure elimination of nonspecific products. The amount of mRNA was determined by comparing with the standard curves and normalization by β-Actin. The primer sequences utilized are listed in Additional file 6: Table S4.

### Western blot analysis

Immunologic detection of Ntrk2 with rabbit polyclonal Ntrk2 antibody (Abcam, ab33655, 1:500 dilution) was performed. In brief, 20 μg total protein was loaded on a 6% polyacrylamide gel, electrophoresed, and transferred to polyvinylidene diflouride membranes, which were blocked with Tween-Tris buffered salt solution (TTBS) containing 5% skim milk. Membranes with incubated overnight at 4^0^ with Ntrk2 antibody. After washing 3 times with TTBS, membranes were incubated with anti-rabbit-IgG-HRP for 1 hour at room temperature and images recorded. β-actin was used as loading control.

### Immunohistochemistry

Ntrk2 immunohistochemistry was performed using standard protocols. In brief, primary anti-Ntrk2 antibody (Abcam, ab33655) diluted 1:200 in the blocking buffer was applied to 10 um thick mouse lung sections and incubated for 30 min. After the slides were rinsed and incubated with the peroxidase-labeled, polymer-conjugated goat anti-rabbit secondary antibody, the labeled structure was visualized.

### Transfection of miR-29 mimics

SiPORT NeoFx Transfection Agent (Ambion) was used to deliver miRNA mimics (small duplex RNAs) into BASC cells according to the manufacturer’s instructions. Cells were plated in six-well dishes at density of 120,000 cells/well. MiRNA mimics including miR-29a mimic, miR-29c mimic and miRNA mimic negative control (Applied Biosystems) were transfected respectively at the final concentration of 25 nM. The sequence for the miR-29a mimic is UAGCACCAUCUGAAAUCGGUUA; for the miR-29c mimic UAGCACCAUUUGAAAUCGGUUA. After 24 hours post-transfection, miRNAs were isolated using the mirVanaTM miRNA Isolation kit. The overexpression of miR-29a and miR-29c were confirmed by Real-time based TaqMan MicroRNA Assay (Applied Biosystems). MiRNA expression levels relative to SnoRNA-202 (internal control) were calculated on the basis of ΔΔCt methods. The n-fold change in miRNAs expression was determined according to the method of 2^−ΔΔCT^.

## Competing interests

The authors declare that they have no competing interests.

## Authors’ contributions

JD, SA, ST, SS carried out biological experiments. GJ, JD, CC and YWA carried out the analysis, GJ implemented the computational method. JD drafted the manuscript. DAW, WAC and YP conceived and directed the project, participated in the design and coordination of the study, and edited the manuscript. All authors read and approved the final manuscript.

## Funding

Dr. Prakash was supported by NIH grants HL056470 and HL088029. Dr. Wigle was supported by an NIH K12 Career Development Award through the Mayo Clinic Cancer Center, and a Career Development Award from the International Society for Heart and Lung Transplantation (ISHLT). The funders had no role in study design, data collection and analysis, decision to publish, or preparation of the manuscript.

## Supplementary Material

Additional file 1: **Figure S1.** Relative miR-29 expression in BASC cells after transfection of miR-29 mimics. Click here for file

Additional file 2: **Table S5.** MiR-29 targeted gene expression in BASC cells following transfection with miR-29 mimics. Click here for file

Additional file 3: **Table S1.** Identification of functionally related clusters in hyperoxia-induced BPD. All 2714 mRNA probes which significantly changed under hyperoxia were classified into functionally related clusters using DAVID. The 6 top functionally related clusters including cell cycle, adhesion, mobility, angiogenesis, taxis, and inflammation are listed. Click here for file

Additional file 4: **Table S2.** List of hyperoxia-responsive genes involved in functional categories of cell cycle, adhesion, mobility, migration, angiogenesis, taxis and inflammation. Potential miRNAs involved in regulation of these functions are also listed. Click here for file

Additional file 5: **Table S3.** Overlap of dynamically regulated miRNAs in normal lung alveolar development and hyperoxia-induced BPD. Among 66 miRNAs identified from O_2_-induced BPD, 33 miRNAs overlapped with the 117 miRNA identified as dynamically regulated during lung organogenesis. The expressed copy number values of all 117 miRNA are listed. Click here for file

Additional file 6: **Table S4.** Primer sequences for real-time PCR. Click here for file
