# Supplementary figures and images for: MicroRNA-mRNA interactions in a murine model of hyperoxia-induced bronchopulmonary dysplasia
Source: BMC Genomics. 2012 May 30;13:204. doi: 10.1186/1471-2164-13-204 (PMC3410783; doi:10.1186/1471-2164-13-204)

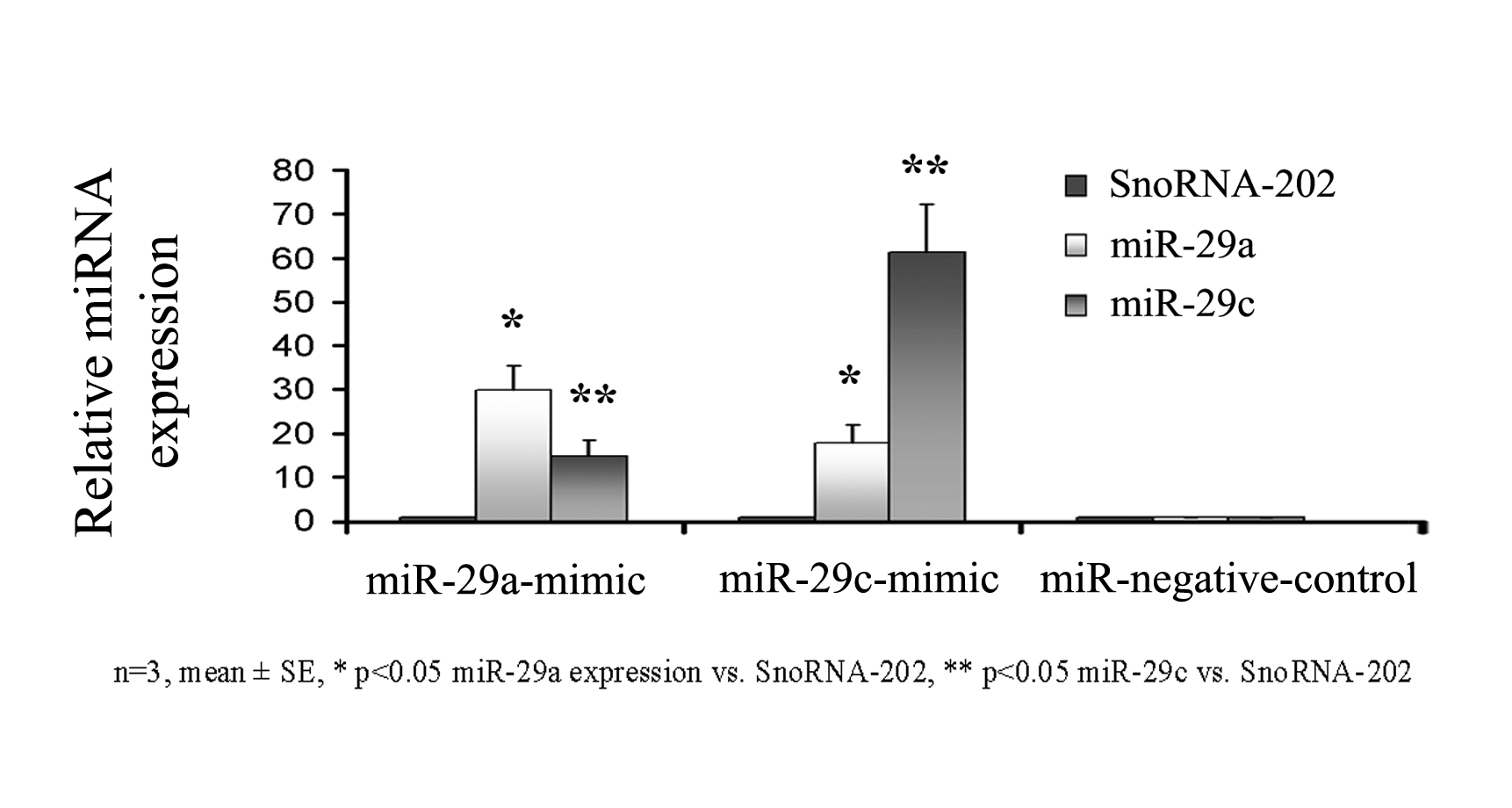

Supplement: Additional file 1: — Figure S1. Relative miR-29 expression in BASC cells after transfection of miR-29 mimics. [file 1471-2164-13-204-S1.tiff]
